# NNMT depletion contributes to liver cancer cell survival by enhancing autophagy under nutrient starvation

**DOI:** 10.1038/s41389-018-0064-4

**Published:** 2018-08-10

**Authors:** Ji Hye Shin, Chang Wook Park, Gyesoon Yoon, Sun Mi Hong, Kwan Yong Choi

**Affiliations:** 1 Department of Life Sciences, Pohang University of Science and Technology, 77 Cheongam-ro, Nam-gu, Pohang, Gyeongbuk 37673 Korea; 2 Biokogen Inc. Korea National Food Cluster #255, 110 Dongchonje-gil, Wanggung-myeon, Iksan, Jeonbuk 54576 Korea; 3 Department of Biochemistry, Ajou University School of Medicine, 164 World cup-ro, Yeongtong-gu, Suwon, Gyeonggi 16499 Korea; 4 Department of Biomedical Science, Graduate School, Ajou University, 164 World cup-ro, Yeongtong-gu, Suwon, Gyeonggi 16499 Korea; 5 Division of Integrative Biosciences and Biotechnology, Pohang University of Science and Technology, 77 Cheongam-ro, Nam-gu, Pohang, Gyeongbuk 37673 Korea

## Abstract

Nicotinamide N-methyl transferase (NNMT) transfers a methyl group from S-adenosyl-L-methionine (SAM) to nicotinamide (NAM), producing 1-methylnicotinamide (1MNA). NNMT has been implicated in several cancer types and recently in metabolism, but its role in autophagy regulation has not yet been investigated. In this study, we determined that NNMT negatively regulated autophagy at the stage of ULK1 activation through protein phosphatase 2A (PP2A) activity. Specifically, NNMT knockdown increased PP2A methylation and subsequently enhanced phosphatase activity. Consequent p-ULK1 (S638) dephosphorylation derepressed ULK1 activity, resulting in autophagy induction. Accordingly, NNMT downregulation rescued tumor cells under nutrient deficiency in vivo, which was alleviated by ULK1 inhibitor treatment. In summary, our results suggest a novel mechanism by which tumor cells protect themselves against nutrient deprivation through NNMT suppression to accelerate autophagy.

## Introduction

Nicotinamide N-methyl transferase (NNMT) is a methyltransferase that transfers a methyl group from S-adenosyl-L-methionine (SAM) to nicotinamide (NAM) to produce S-adenosylhomocysteine and 1-methylnicotinamide (1MNA) in the methionine cycle and is one of the key effectors of global methylation status in the cellular metabolome^1^. Since NNMT utilizes the NAD+ precursor NAM and the methyl donor SAM, its activity may affect two enzyme types: NAD+-dependent enzymes and SAM-dependent methyltransferases^2^. According to microarray and proteomic data, NNMT is highly expressed in various cancer types, including renal clear cell carcinoma, bladder cancer, gastric cancers, and colorectal cancers^3^. Recent papers have highlighted the metabolic function of NNMT, which is beneficial for the host in the liver but unfavorable in adipose tissue^4,5^. Given this contradictory role in physiology and disease, further investigation in terms of metabolism and cancer is warranted^6^.

Autophagy is an ancient and highly evolutionarily conserved catabolic process that targets proteins and organelles within the cell for lysosomal degradation^7^. While basal autophagy is generally present in cells, autophagy can be strongly activated in response to a variety of stresses, such as hypoxia and nutrient starvation^8^. Once activated, a series of protein complexes composed of autophagy-related gene (ATG) products orchestrate the formation of double membrane vesicles called autophagosome that engulf cytoplasmic cargo, including proteins, organelles, and autophagy receptors such as sequestasome1 (p62)^9^. Among ATG proteins, ULK1 (ATG1) is a serine/threonine kinase and the most upstream component of the core autophagy machinery^10^. ULK1 is regulated by mammalian target of rapamycin complex 1 (mTORC1) and AMP-activated protein kinase (AMPK). mTOR inhibits autophagy via ULK1 phosphorylation at serines 638 and 758^11^. AMPK binds ULK1 directly, leading to ULK1 phosphorylation and activation^12^. The AMPK sites in ULK1 include S556, S638, and T660. Depending on the context, autophagy promotes tumor cell survival or conversely suppresses tumorigenesis. Autophagy suppresses tissue damage, chronic inflammation, DNA damage response, and genome instability, which are known to initiate cancer^13^. Therefore, autophagy may suppress liver and pancreatic tumor initiation by limiting tissue damage. Paradoxically, much evidence also supports a role for autophagy in maintaining tumor cell survival under metabolic stress^14^. Recent studies have also shown that autophagy induction causes resistance to multiple standard chemotherapeutic agents, such as paclitaxel^15^. In addition, autophagy has been implicated in the survival of dormant tumor cells and, more importantly, may be critical for renewed growth of these tumor cells. These contradictory autophagy roles contribute to tumor cell survival or tumor suppression, acting as a double-edged sword in cancer^16^.

PP2A is one of the main serine/threonine phosphatases in mammalian cells and maintains cell homeostasis by counteracting most of the kinase-driven intracellular signaling pathways^17^. PP2A must be activated before being assembled into an active holoenzyme, which is a heterotrimeric complex comprising one catalytic domain, one scaffold subunit, and one of many possible regulatory subunits^18^. PP2Ac undergoes post-translational modification on its unstructured carboxy-terminal tail, phosphorylation on T304 and Y307, and methylation on L309^19^. Especially, the methylation is reversibly controlled by a PP2A-specific methyltransferase known as leucine carboxy methyltransferase (LCMT-1) and by PP2A-specific methylesterase 1 (PME-1)^20,21^. Since PP2A methylation is essential for cell function, cells will suffer apoptosis in the absence of LCMT-1^22^. PP2A has been reported to dephosphorylate over 300 substrates due to the diversity of its holoenzyme structure^23^. As most of these substrates are involved in cell cycle regulation, the majority of PP2A-mediated dephosphorylation events play a negative regulatory role in proliferation. Conversely, PP2A-mediated dephosphorylation has occasionally been shown to positively regulate proliferation pathways. PP2A is implicated in a variety of human diseases due to its important function in the cell cycle and many other essential cellular processes^24^. Accordingly, through several molecular strategies, its inactivation has been described as a common event in cancer cells.

In this study, we suggest a novel mechanism by which NNMT negatively regulates autophagy progression in liver cancer. NNMT depletion enhances PP2A methylation and activation, resulting in ULK1 derepression and autophagy progression. We also demonstrate that NNMT knockdown (KD) tumors are dependent on autophagy for survival and growth, and thus autophagy inhibitor treatment is detrimental to the tumors. Because NNMT is suggested to be downregulated in liver cancer, use of autophagy inhibitors as therapeutics may be worthwhile.

## Results

### NNMT KD enhances autophagy in liver cancer cells

**Fig. 1:**
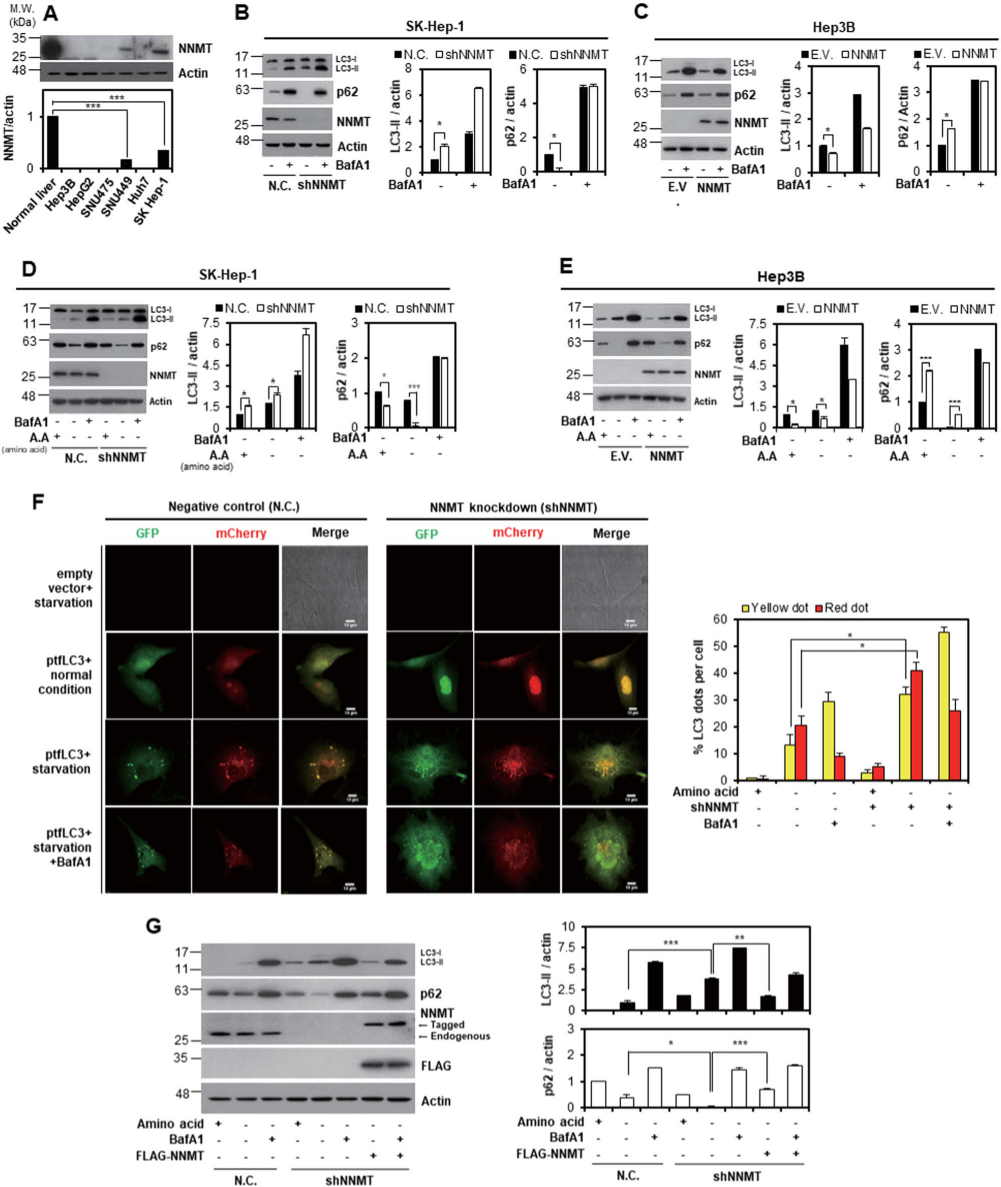
NNMT negatively regulates autophagy in liver cancer cells. **a** NNMT expression in normal tissue and various liver cancer cell lines was assessed by western blot analysis using whole-cell lysates. The NNMT protein level was analyzed by densitometry using ImageJ software. The relative ratio of NNMT levels was normalized to actin expression. **b**–**e** p62 and LC3-II levels were assessed by western blotting in SK-Hep-1-N.C., SK-Hep1-shNNMT, Hep3B-E.V., and Hep3B-NNMT OE cells. **b**–**c** Cells were cultured in growth medium for 120 h, followed by treatment with or without 10 nM BafA1 for an additional 4 h. **d**–**e** Cells were incubated under amino acid starvation (HBSS) for 4 h in the absence or presence of 10 nM BafA1. p62 and LC3-II levels were detected by western blot analysis. **f** SK-Hep-1-shNNMT cells were transiently transfected with either empty vector (E.V.) or green fluorescent protein (GFP)-mCherry tandem fluorescence-tagged LC3 plasmid for 44 h and exposed to amino acid starvation for an additional 4 h with or without 10 nM BafA1 treatment. Confocal microscopy images for autophagosome/autolysosome analysis. Scale bar indicates 10 μm. The histogram represents the % of LC3 puncta per cell, with yellow (autophagosome)/red dots (autolysosomes). Each bar represents the mean ± SD. **g** SK-Hep-1-shNNMT cells were transfected with plasmid expressing FLAG-tagged NNMT. After transfection for 44 h, cells were incubated under amino acid starvation for 4 h in the absence or presence of 10 nM BafA1. The expression level of LC3-II or p62 were quantified by ImageJ software. Bar graph represents the normalized band intensities of each protein to actin. The histogram bars represent the mean ± SD of three independent experiments (*p < 0.05, **p < 0.01, ***p < 0.001)

In a previous report of a genome-wide siRNA screen of autophagy regulators, NNMT KD was shown to increase autophagy flux^25^. Since NNMT is abundant in the liver and implicated in several tumor types^2^, we decided to investigate the role of NNMT in autophagy signaling in liver cancer. First, we assessed endogenous NNMT expression levels in normal liver tissue and cancer cell lines. Notably, the NNMT level was dramatically downregulated in liver cancer cells. Among the liver cancer cell lines, NNMT levels were higher in SK-Hep-1 and SNU-449 cells than in Hep3B or HepG2 cells (Fig. 1a). Therefore, we established SK-Hep-1 cells stably expressing negative control shRNA (SK-Hep-1-N.C.) or NNMT shRNA (SK-Hep-1-shNNMT). We also obtained Hep3B cells stably expressing empty vector (Hep3B-E.V.) or NNMT overexpression (OE) vector (Hep3B-NNMT OE) as model cell lines to study NNMT function. Using the model cell lines, we measured autophagic flux by examining p62 and LC3B-II levels in the presence or absence of Bafilomycin A1 (BafA1), which blocks degradation of autophagy substrates. The LC3-II level was increased by 3-fold, and the p62 level was significantly decreased in SK-Hep-1-shNNMT cells compared with that in SK-Hep-1-N.C. cells even without nutrient deprivation (Fig. 1b), indicating that NNMT KD increased autophagy flux in liver cancer cells. In contrast, NNMT OE reduced the LC3-II level and induced p62 accumulation (Fig. 1c). Our data indicate that the NNMT expression level was inversely correlated with autophagy progression in liver cancer cells.

To further confirm the role of NNMT in autophagy, we examined autophagy flux under amino acid starvation, which is a well-known autophagy inducer. Consistent with Fig. 1b–c, NNMT KD significantly accelerated (Fig. 1d) while NNMT OE slowed autophagy flux upon amino acid starvation (Fig. 1e). We also monitored autophagosome and autolysosome formation using mCherry-GFP-LC3 vector. SK-Hep-1-shNNMT cells showed much more abundant autophagosomes (yellow dot) and autolysosomes (red dot) (Fig. 1f). Moreover, levels of autophagosome (yellow dot) were higher in NNMT KD cells with BafA1 treatment than negative control cells with BafA1 treamtent, indicating that NNMT KD enhanced autophagy flux. Lastly, when we reintroduced NNMT into SK-Hep-1-shNNMT cells, p62 accumulated and the LC3-II level was sharply reduced (Fig. 1g). Our results suggest that NNMT negatively regulates the autophagy process in liver cancer cells.

### NNMT negatively regulates ULK1 and autophagy

**Fig. 2:**
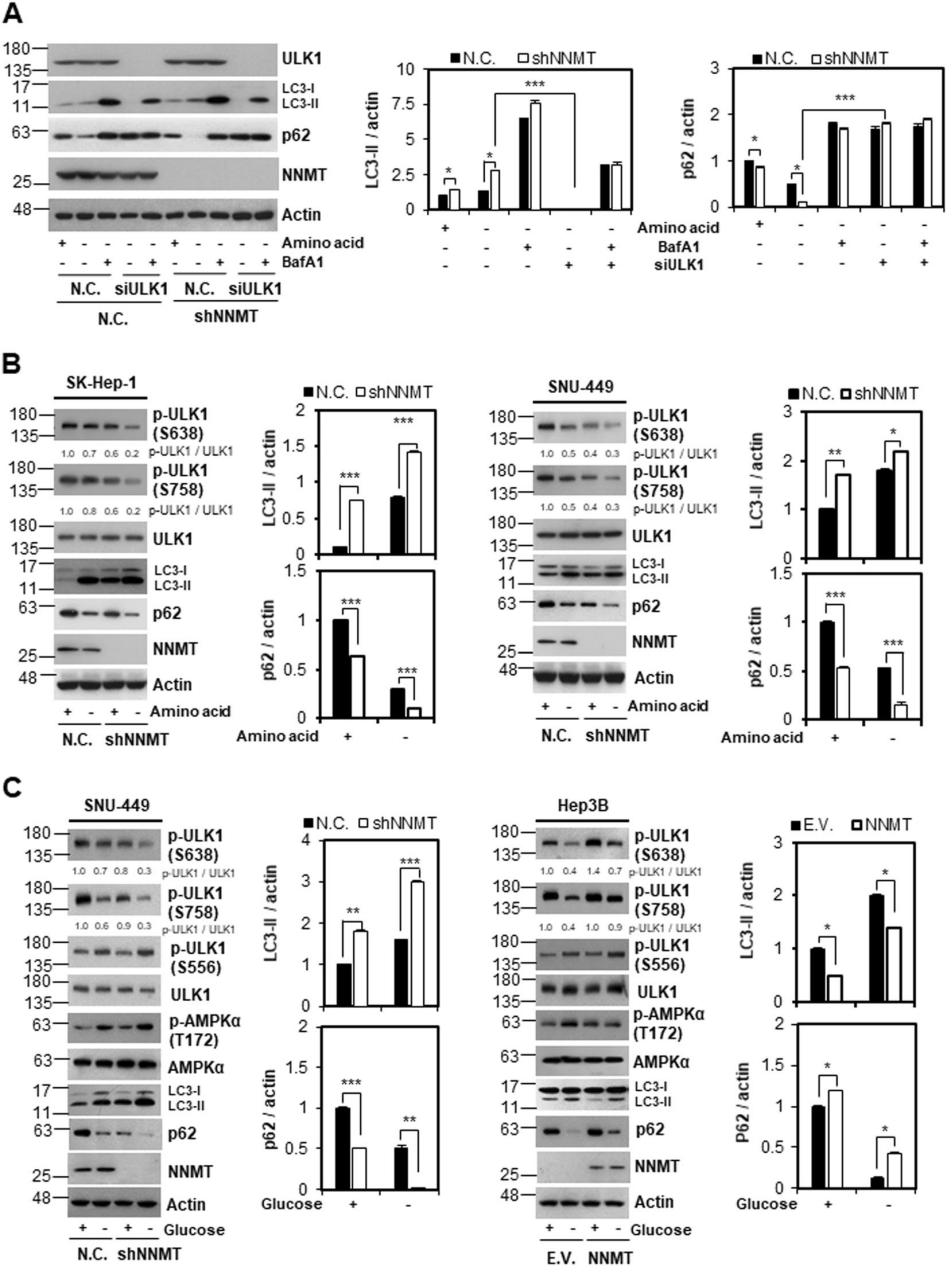
NNMT depletion enhances autophagy and derepresses ULK1 upon nutrient starvation in liver cancer cells. **a** Levels of the indicated proteins were analyzed by western blot in SK-Hep-1-N.C. and SK-Hep-1-shNNMT cells. Cells were transfected with non-targeting (negative control, N.C.) or ULK1 siRNAs for 44 h, followed by amino acid starvation with or without 10 nM BafA1 for an additional 4 h. **b** Western blot analysis of the indicated proteins in SK-Hep-1 and SNU-449 cells cultured in HBSS for 4 h. **c** Western blot analysis of the indicated proteins in SNU-449 cells cultured under glucose deprivation for 18 h and in Hep3B cells cultured for 12 h. Using ImageJ software, the band intensities of LC3-II or p62 were quantified. Each band intensity was normalized to that of actin. The histogram bars represent the mean ± SD of three independent experiments (*p < 0.05, **p < 0.01, ***p < 0.001)

Given the inverse correlation between NNMT expression and autophagy progression, we investigated the underlying mechanism of autophagy regulation by NNMT. Since ULK1 initiates the autophagy in response to various stresses, including nutrient starvation, we examined whether ULK1 could mediate the function of NNMT in autophagy regulation. When we depleted ULK1 using siRNA, p62 accumulated even with a lack of amino acids (Fig. 2a). Notably, NNMT KD did not enhance p62 degradation and autophagosome formation in the absence of ULK1 (Fig. 2a and Supplementary Fig. 1), indicating that NNMT KD accelerates autophagy flux upon amino acid deprivation via ULK1.

ULK1 activity is regulated by a reversible phospho-switch^26^. Under normal conditions, S638 and S758 of ULK1 are phosphorylated by mTOR, resulting in autophagy inhibition^11^. Meanwhile, AMPK is activated and ULK1 is phosphorylated at S556 upon glucose starvation, leading to autophagy induction. Therefore, we examined the phosphorylation status of ULK1 in response to amino acid starvation under NNMT KD conditions. NNMT KD remarkably downregulated p-ULK1 (S638) and p-ULK1 (S758) levels under amino acid deprivation, increasing of autophagy flux (Fig. 2b). Interestingly, ULK1 was dephosphorylated even without starvation when NNMT was depleted. In contrast to dephosphorylation of S638 and S758, phosphorylation at S556 was not affected by NNMT expression (Fig. 2c). Irrespective of NNMT, the p-ULK1 (S556) level was increased by AMPK upon glucose starvation, which induced autophagy. These results suggest that NNMT might be involved in ULK1 dephosphorylation at S638 and S758.

### NNMT KD enhances autophagy via PP2A-mediated ULK1 activation

**Fig. 3:**
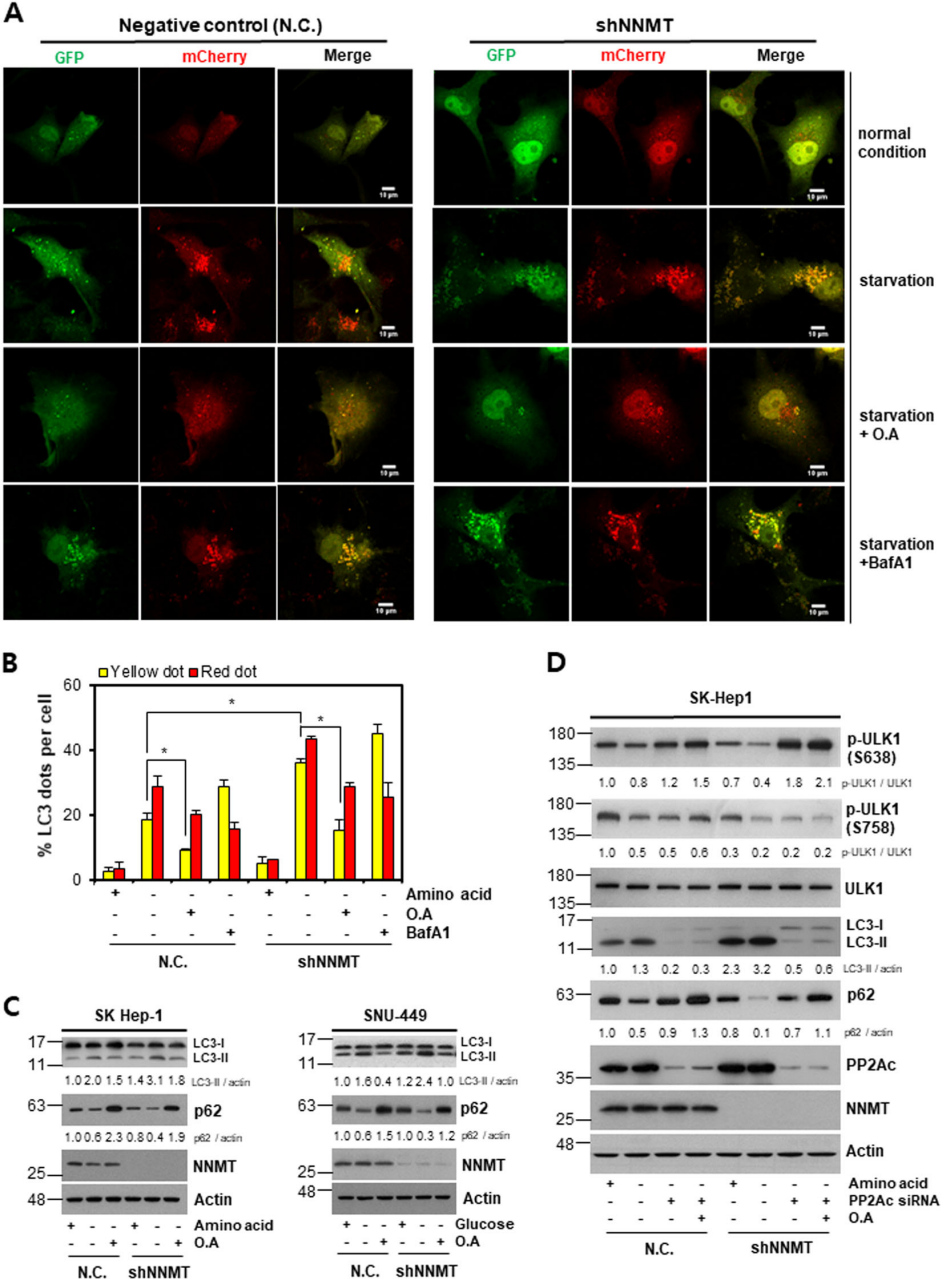
NNMT knockdown elevates autophagy response via PP2A-mediated ULK1 activation. **a**–**b** SK-Hep-1-N.C. and SK-Hep-1-shNNMT cells were transiently transfected with either empty vector (E.V.) or GFP-mCherry tandem fluorescence-tagged LC3 plasmid for 44 h, followed by treatment with or without 10 nM okadaic acid for 2 h and/or subjection to amino acid starvation for an additional 4 h in the presence or absence of 10 nM BafA1 treatment. **a** Confocal microscopy images for autophagosome/autolysosome analysis. Scale bar indicates 10 μm. **b** The histogram represents the % of LC3 puncta per cell, with yellow (autophagosome)/red dots (autolysosomes). The data are shown as the mean ± SD and are representative of three independent experiments (*p < 0.05). **c** Western blot analysis of autophagy markers in shNNMT cells. *Left panel*: SK-Hep-1 cells were treated with or without 10 nM okadaic acid for 6 h and subjected to amino acid starvation for an additional 6 h. *Right panel*: SNU-449 cells were exposed to glucose starvation for 6 h, followed by treatment with or without 10 nM okadaic acid for an additional 12 h. **d** Western blot analysis of autophagy markers in SK-Hep-1-N.C. and SK-Hep-1-shNNMT cells. The cells were transiently transfected with N.C. or PP2Ac siRNA for 36 h, followed by treatment with or without 10 nM okadaic acid for 6 h. Then, cells were challenged with amino acid starvation for an additional 6 h, followed by western blot. The indicated proteins were quantified using ImageJ software. Each band intensities were normalized to that of actin. The data are representative of three independent experiments

According to Fig. 2, we speculated that the enhancement of autophagy by NNMT KD could be due to ULK1 dephosphorylation. Supporting our assumption, Wong et al. showed that PP2A could remove the phospho-group from ULK1 and reactivate it^27^. Therefore, we treated cells with okadaic acid (O.A), a pharmacological PP2A inhibitor. O.A treatment significantly suppressed autophagosomes and autolysosomes irrespective of NNMT status under amino acid deprivation (Fig. 3a, b). Furthermore, the LC3-II level was reduced and the p62 level was restored under O.A treatment, reflecting autophagy inhibition, in spite of NNMT KD and nutrient depletion through either amino acid or glucose starvation (Fig. 3c). These data suggest that PP2A enhances the autophagy induced by NNMT KD. We further examined whether PP2A could be a downstream effector of NNMT in autophagy regulation using siRNA targeting the catalytic subunit of PP2A (PP2Ac). Similar to O.A treatment, PP2Ac KD dramatically blocked autophagy even in the absence of NNMT under amino acid deprivation (Fig. 3d). Notably, PP2A KD with or without O.A treatment restored the p-ULK1 (S638) level but not the p-ULK1 (S758) level in SK-Hep-1-shNNMT cells. Collectively, these data suggest that NNMT affects PP2A to modulate ULK1 phosphorylation status and consequently autophagy progression.

### NNMT KD increases PP2A methylation and consequently phosphatase activity

**Fig. 4:**
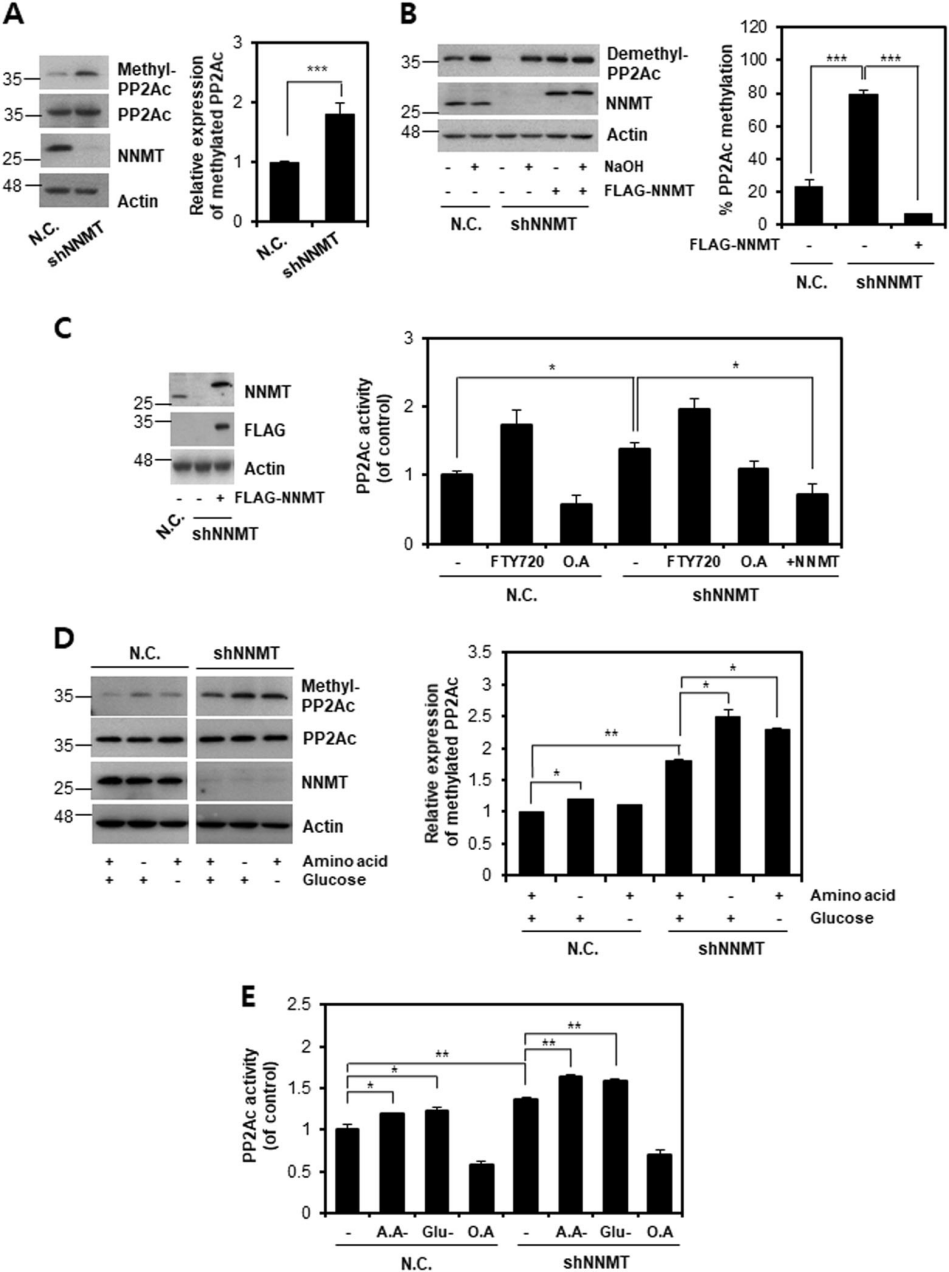
NNMT negatively regulates PP2A methylation and activity in liver cancer cells. **a** SK-Hep-1-N.C. and SK-Hep-1-shNNMT cells were incubated in growth medium for 60 h, followed by western blot analysis using methyl-PP2Ac antibody. The methylated level of PP2Ac was obtained via densitometry using ImageJ software and were normalized to that of actin. **b** SK-Hep-1-shNNMT cells were transfected with E.V or FLAG-NNMT vector for 48 h, followed by an in vitro methylation assay; cell lysates were incubated with NaOH to remove all methyl groups from the proteins, followed by western blot analysis using a demethyl-PP2Ac antibody. The band intensities were analyzed via densitometry using ImageJ software, and the percentage of methylated PP2Ac was determined by subtracting the neutralized lysates with NaOH (+) from lysates without NaOH (−) in each group. **c** SK-Hep-1-N.C./SK-Hep-1-shNNMT cells were treated with 1 µM FTY720 for 12 h or 10 nM okadaic acid (O.A) for 6 h, respectively. SK-Hep-1-shNNMT cells were transfected with a FLAG-NNMT vector for 48 h. Western blotting was performed to examine the efficiency of the plasmid expressing NNMT in SK-Hep-1-shNNMT cells (left panel). A PP2A activity assay was conducted (right panel). The bars represent PP2A activity, which is shown as the fold change relative to the control. **d**–**e** SNU-449-N.C. and SNU-449-shNNMT cells were incubated under glucose deprivation for 6 h or amino acid starvation for 1 h, followed by measurement of PP2Ac methylation or activity. **d** Western blot analysis using a methyl-PP2Ac antibody. The band intensities shown below each band were obtained via densitometry using ImageJ software and were normalized to that of actin, which was used as the loading control. **e** The bars represent PP2A activity, which is shown as the fold change relative to the control. Each histogram bar represents the mean ± SD of three independent experiments (*p < 0.05, **p < 0.01, ***p < 0.001)

Since we found that PP2A might mediate the effect of NNMT on ULK1 regulation to accelerate autophagy, we investigated the detailed mechanism of PP2A regulation by NNMT. PP2A phosphatase activity was reported to be increased by methylation on its catalytic site^28^. NNMT OE was also shown to reduce PP2A methylation, but NNMT KD increased the methylated-PP2A level^29^. Therefore, we assumed that NNMT downregulation increased PP2A phosphatase activity by increasing methylation, consequently enhancing ULK1 dephosphorylation and inducing autophagy. To test this assumption, we assessed the methylation status of PP2A in SK-Hep-1-shNNMT cells and found that PP2A methylation was increased by NNMT KD (Fig. 4a). Moreover, we examined the methylated-PP2A levels using demehtyl-PP2Ac antibody prior to and after NaOH treatment to remove methyl group. As expected, demethylated-PP2A was dramatically decreased in SK-Hep-1-shNNMT cells, confirming that NNMD KD increased PP2A methylation (Fig. 4b). However, when we reintroduced a FLAG-NNMT vector to SK-Hep-1-shNNMT cells, the demethylated-PP2A level dramatically increased, suggesting that NNMT could negatively regulate PP2A methylation. Next, we investigated whether NNMT could negatively control the phosphatase activity of PP2A. As expected, FTY720, a PP2A activator, enhanced PP2A activity, which was inhibited by O.A treatment (Fig. 4c). Moreover, PP2A activity was increased by NNMT KD, but this effect was reversed by re-expression of NNMT. Last, to elucidate the connection between NNMT status and PP2A activation in autophagy regulation, we measured PP2A methylation and activity under nutrient starvation, such as amino acid and glucose starvation. Nutrient starvation led to PP2A methylation (Fig. 4d) and activation (Fig. 4e), which was intensified by NNMT KD. Collectively, these data suggest that NNMT KD increases PP2A methylation and consequently enhances phosphatase activity in the absence or presence of nutrients, including amino acid and glucose.

### NNMT KD enhances liver tumor growth and survival under nutrient starvation in vitro and in vivo

**Fig. 5:**
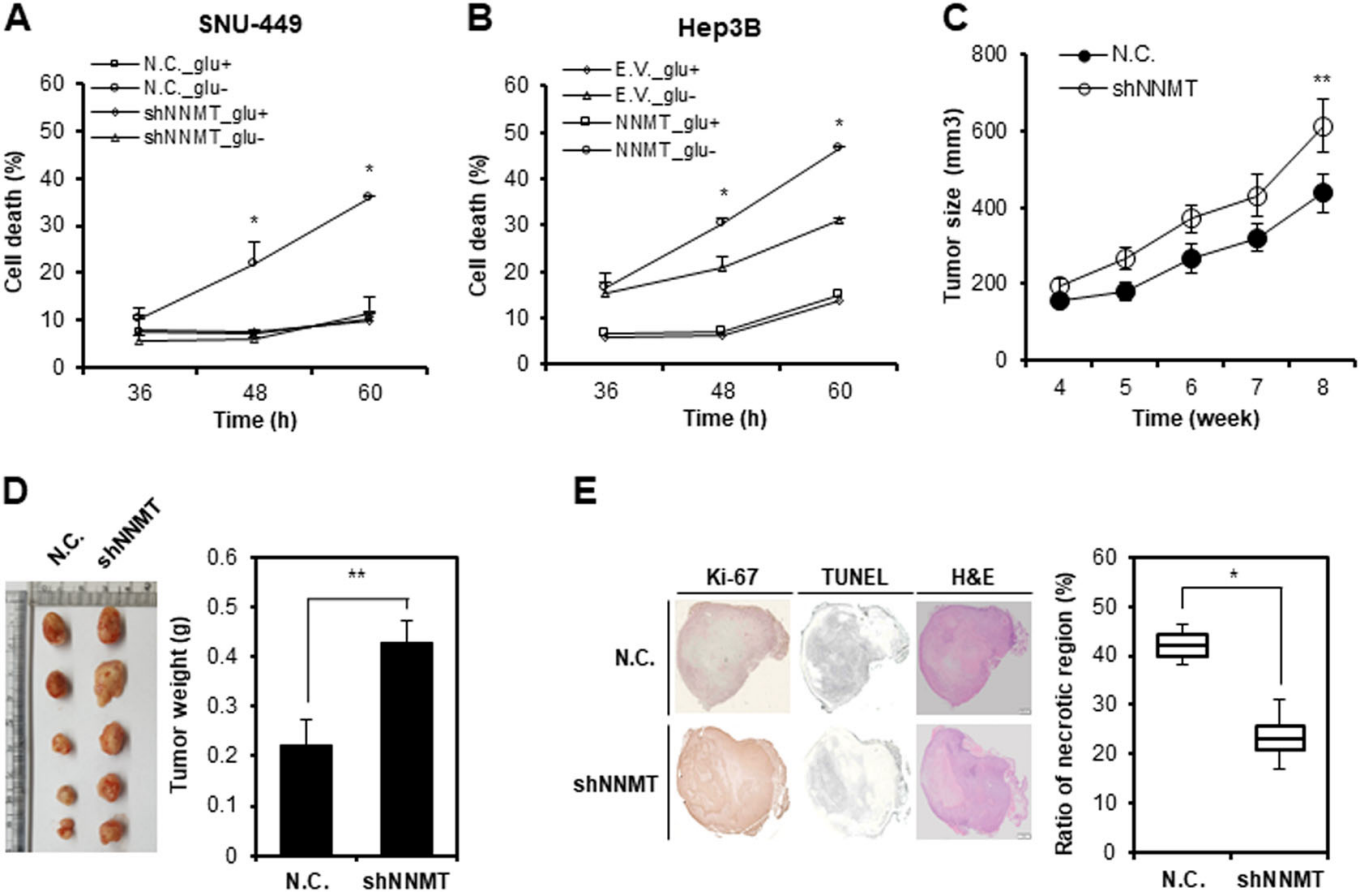
NNMT silencing enhances liver tumor growth and resistance to nutrient starvation in vitro and in vivo. **a**–**b** SNU-449-shNNMT cells and Hep3B-NNMT OE cells were cultured under glucose starvation for the indicated times. Cell death was assessed with a trypan blue assay. The data shown are representative of three independent experiments. Each histogram bar represents the mean ± SD of three independent experiments (*p < 0.05). **c**–**d** SK-Hep-1-N.C. and SK-Hep-1-shNNMT cells (1 × 10^7^) were injected subcutaneously into mice. **c** Four weeks after the injection, the average tumor size was determined every week. The value represents the tumor volume mean ± SD (**p < 0.01). **d** A representative image of tumors obtained from the xenograft experiment. At 8 weeks after the injection, mice were sacrificed, and their tumors were weighed. Each tumor weight represents the mean ± SD (**p < 0.01). **e** Immunohistochemical staining of xenograft tumor tissues induced through injection of each cell group (*n* = 5); the tumor tissues were subjected to hematoxylin and eosin (H&E), Ki-67, or TUNEL staining. Representative images of the staining at a magnification of 10×. Scale bars indicate 500 μm for N.C. and 1 mm for shNNMT cells, respectively. The histogram bar represents necrotic region % assessed via the TUNEL assay and analyzed using ImageJ software. Horizontal lines: group medians, Boxes: 25–75% quartiles. Vertical lines: range, maximum and minimum (*p < 0.05)

To clarify the role of NNMT-regulated autophagy in tumor cells, we subjected model cell lines to glucose starvation for 60 h. Since NNMT was expressed in SNU-449 cells and not in Hep3B cells, we established SNU-449 cells stably expressing negative control shRNA (SNU-449-N.C.) or NNMT shRNA (SNU-449-shNNMT), which were subjected to glucose starvation, and we found that SNU-449-N.C. cells underwent cell death over time (Fig. 5a). However, SNU-449-shNNMT cells were resistant to cell death under glucose starvation, which was comparable to cell death under the glucose-rich conditions (Fig. 5a and Supplementary Fig. 2). Hep3B cells stably expressing empty vector (Hep3B-E.V.) or NNMT OE vector (Hep3B-NNMT OE) were incubated in the absence of glucose, and cell death was assessed. In contrast with NNMT KD, NNMT OE increased the sensitivity to glucose starvation in Hep3B cells (Fig. 5b). These data indicated that NNMT downregulation enables tumor cells to survive under nutrient-deficient conditions. To further evaluate the effect of NNMT downregulation in vivo, we introduced SK-Hep-1-shNNMT cells into nude mice via subcutaneous injection and measured the tumor size for 4 weeks. As expected, NNMT KD remarkably promoted tumor growth in vivo (Fig. 5c). Consequently, tumors with NNMT KD were 28% bigger and weighed 46% more than negative control tumors, when tumor tissues were extracted (Fig. 5d). Finally, we analyzed the tumor tissues using immunohistochemistry. The area of necrotic regions, in which most cells were dead due to the distance from the blood vessels and the lack of nutrients, was 54% smaller in tumor tissues with NNMT KD than in the negative control tumor tissues (Fig. 5e). Collectively, these data suggest that NNMT downregulation might be beneficial to tumor growth and survival under harsh conditions, such as nutrient deprivation.

### NNMT KD renders liver tumor cells vulnerable to autophagy inhibitor treatment

**Fig. 6:**
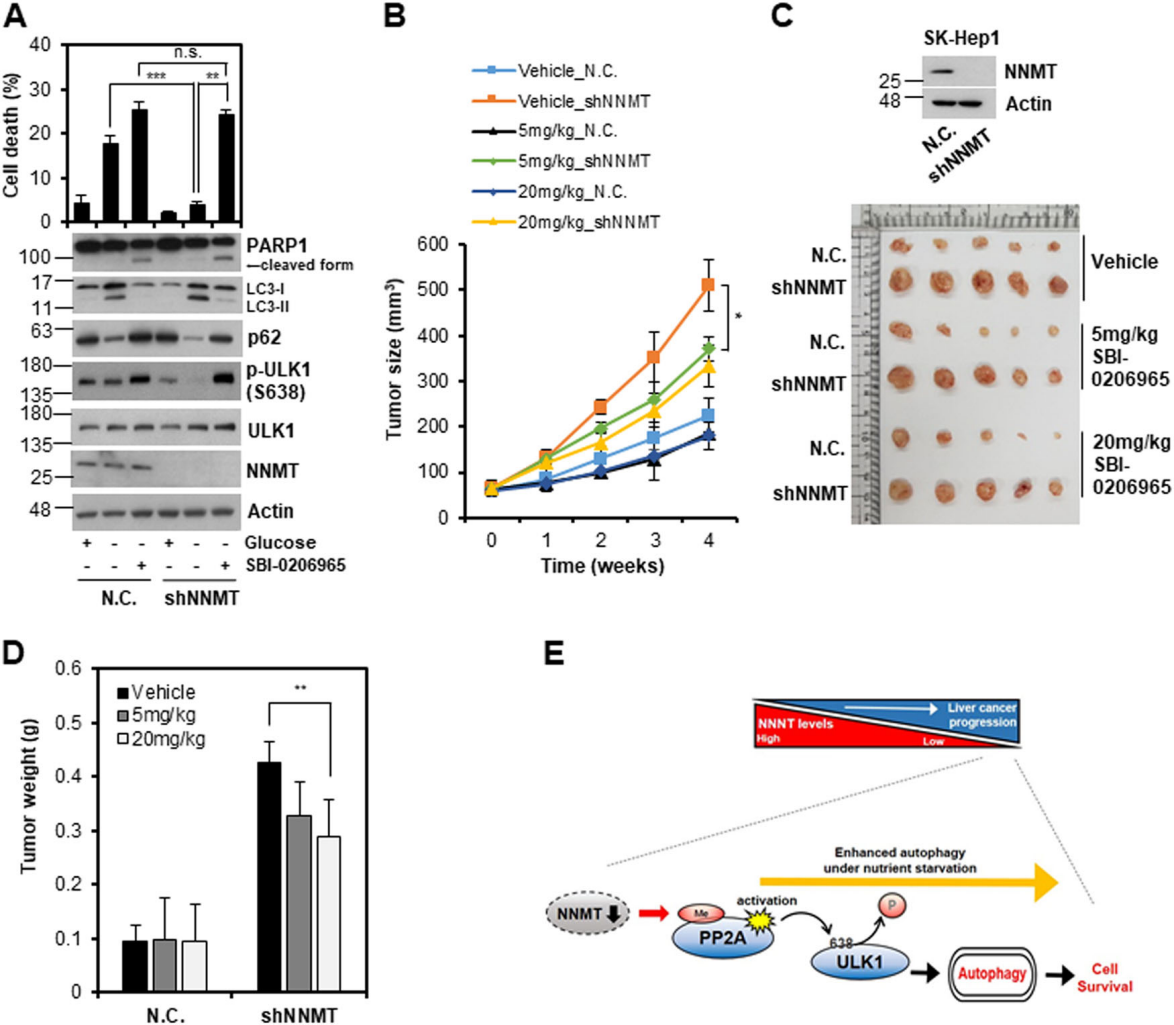
Liver cancer cells with NNMT knockdown exhibit increased sensitivity to autophagy inhibitor. **a** SNU-449-N.C. and SNU-449-shNNMT cells were pretreated with or without 10 µM SBI-0206965 for 12 h and/or subjected to glucose starvation for an additional 36 h. Cell death was assessed with a trypan blue assay (upper), and western blotting analysis of the indicated proteins was performed (lower). Each histogram bar represents the mean ± SD of three independent experiments (**p < 0.01, ***p < 0.001, n.s. not significant). **b**–**d** SK-Hep-1-N.C. and SK-Hep-1-shNNMT cells (1 × 10^7^) were injected subcutaneously into mice. After 14 days, mice with a tumor size >50 mm^3^ were randomized blindly (*n* = 5 for each treatment group). Vehicle or SBI-0206965 (5 or 20 mg/kg) was then injected intraperitoneally into mice three times a week for 4 weeks. **b** Tumor sizes were measured in five mice each week after the injection of cells, and the presented value represents the tumor volume mean ± SD (*p < 0.05). **c** Tumors were collected from the sacrificed mice 6 weeks after tumor cell injection, and tumor size in each group was compared. **d** Tumor weights were measured after mice were sacrificed, and each tumor weight value represents the mean ± SD (**p < 0.01). **e** Schematic diagram of autophagy regulation by NNMT in liver cancer progression

Since we demonstrated that NNMT KD promoted tumor growth and survival via autophagy acceleration in vivo, we examined whether tumor cells with NNMT KD were susceptible to autophagy inhibitor treatment. We found that 18% of SNU-449-N.C. cells were dead in response to glucose starvation, but SNU-449-shNNMT cells showed tolerance to starvation, with only 4% cell death (Fig. 6a). When we treated SNU-449-N.C. cells with SBI-0206965, a ULK1 inhibitor^30^, cell death was increased by 1.4-fold under glucose starvation. However, SBI-0206965 treatment dramatically increased cell death by 6-fold (from 4 to 24.3%) in SNU-449-shNNMT cells under glucose starvation. Notably, SNU-449-shNNMT cells were vulnerable to the ULK1 inhibitor due to autophagy inhibition. O.A treatment also induced sensitivity to glucose deprivation in NNMT KD cells in similar to ULK1 inhibitor in vitro (Supplementary Fig. 3). To confirm that autophagy inhibitor treatment could slow the growth of tumors with NNMT depletion, we subcutaneously injected SK-Hep-1-N.C. or SK-Hep-1-shNNMT cells into mice and treated them with SBI-020695 for 4 weeks. Negative control tumors shrunk by 20% in response to 20 mg/kg of SBI-020695. However, the size of tumors with NNMT KD was reduced by 34% upon drug treatment (Fig. 6b). Moreover, the weight change of negative control tumors was independent of the autophagy inhibitor, but the inhibitor drastically reduced the weight of tumors with NNMT KD by 32% (Fig. 6d). Collectively, our data demonstrate that NNMT downregulation offers survival advantages to tumor cells under nutrient deprivation via autophagy promotion, but an autophagy inhibitor can reverse the advantage. Therefore, an autophagy inhibitor might be a therapeutic strategy for treatment of tumors with NNMT downregulation, which can survive even in the absence of nutrients.

## Discussion

NNMT is expressed at the highest levels in liver tissues^1^ and has recently gained attention as a metabolic regulator in the liver^4^. In addition, through genome-wide analysis, the NNMT gene was predicted to be an autophagy regulator^25^. NNMT has been shown to be overexpressed in various cancer types, including gastric cancer, colorectal cancer, and oral squamous cell carcinoma, but was notably repressed in liver cancer^2,31^. Therefore, we investigated the role of NNMT in autophagy regulation in liver cancer cells. Consistently, we confirmed that NNMT expression was highly repressed (Fig. 1a). Although some types of cancer cells showed NNMT expression, it was negligible compared with normal liver tissue. Accordingly, we established model cell lines with stable NNMT KD or OE (Fig. 1b, c). NNMT KD increased autophagy flux, and NNMT OE slowed it. Even though NNMT affected autophagy under fed conditions, the NNMT effect was more distinct under starvation conditions (Fig. 1d–g). Moreover, when NNMT was reexpressed in NNMT KD cells, NNMT KD-mediated autophagy acceleration was slowed. In conclusion, we clearly demonstrated that NNMT acts as a negative regulator of autophagy under normal conditions and nutrient poor conditions.

ULK1 is the most upstream component of the autophagic signaling pathway and regarded as the master regulator of autophagy^32^. Hence, we investigated whether NNMT-mediated autophagy regulation could be mediated by ULK1. When ULK1 was depleted, autophagy flux was suppressed despite NNMT KD, indicating that ULK1 might be a downstream effector of NNMT in autophagy regulation (Fig. 2a). The enzymatic activity of ULK1 is regulated by phosphorylation and dephosphorylation^26^. mTORC1 phosphorylates ULK1 at S638 and S758, inhibiting ULK1 activity and leading to autophagy suppression^11^, whereas S556 is the target site of AMPK and phosphorylated upon glucose starvation, which activates ULK1, thus initiating autophagy. We found that NNMT KD resulted in a remarkable reduction in phosphorylation at S758 and S638 under nutrient deprivation, leading to autophagy acceleration (Fig. 2b). Meanwhile, S638 has been indicated as a substrate for both AMPK and mTOR^33^. However, we did not observe a correlation between AMPK activity and ULK1 phosphorylation at S638, and only S556 phosphorylation was evident under our experimental conditions (Fig. 2c). Moreover, the p-ULK1 (S556) level was not changed by NNMT expression, suggesting that NNMT-mediated autophagy regulation is independent of the AMPK signaling pathway. These results support the notion that S638 phosphorylation occurs irrespective of AMPK activation but is directly proportional to the NNMT expression level.

Since the reduction in ULK1 phosphorylation (S758 and S638) was greater under starvation conditions with NNMT KD than under starvation only, we assumed that NNMT might modulate the phosphatase that regulates ULK1 phosphorylation status. Indeed, PP2A has been reported to dephosphorylate ULK1 at S638 and to induce autophagy^27^. Additionally, the PP2A inhibitors O.A and rifampicin have been shown to block starvation-induced autophagy, strongly suggesting that PP2A can promote autophagy^34,35^. Consistently, we demonstrated that O.A treatment reduced the NNMT KD-mediated autophagy enhancement under glucose starvation as well as amino acid starvation (Fig. 3a–c). Accordingly, phospho-ULK1 (S638) was recovered in response to PP2Ac KD, even in the absence of NNMT and amino acids (Fig. 3d). Furthermore, PP2A KD did not affect phosphorylations of S6 ribosomal protein (mTORC1 substrate) and AMPK (Supplementary Fig. 4). These results support the idea that PP2A might be a downstream effector of NNMT to regulate ULK1 in the autophagy process independent of mTOR and AMPK. However, p-ULK1 (S758) was not changed by PP2Ac KD, suggesting that there might be another phosphatase involved in ULK1 dephosphorylation, in line with a recent study^27^. Meanwhile, it was reported that protein phosphatase 1D magnesium-dependent delta isoform (PPM1D) can also remove the inhibitory ULK1 phosphorylation at S638 in a p53-dependent manner upon genotoxic stresses^36^. However, in all the model cell lines we used, including SK-Hep-1 (wild-type p53), Hep3B (p53 null), and SNU-449 (mutant p53), NNMT KD accelerated autophagy flux upon nutrient starvation. In addition, Torii et al. suggested the possible involvement of other phosphatases than PPM1D in starvation-induced autophagy, due to the reduction of p-ULK1 (S638) independent on the presence of PPM1D. Therefore, our results clearly demonstrate that NNMT KD could induce ULK1 dephosphorylation at S638 via PP2A to accelerate autophagy flux under nutrient starvation conditions.

The enzymatic function of NNMT has a dual role in cellular metabolism. First, it converts NAM to 1MNA, which sequesters NAM and excretes it from the cell. Due to fast equilibration rates of NAM across the plasma membrane and marginal changes in steady-state intracellular NAM levels, it is not feasible that NNMT could regulate NAM levels and consequently affect NAD+-dependent enzymes in all aspects of metabolism, including autophagy regulation^2^. Second, NNMT acts as a methyl donor sink^29^. Since NNMT consumes SAM to methylate NAM and produce 1MNA, NNMT inhibition enhances methylation potential^3^. NNMT silencing increased the availability of methyl groups for LCMT-1 to methylate the C-terminal residue of PP2A for activation^37^. O.A, a well-known inhibitor of PP2A, has also been demonstrated to inhibit PP2Ac methylation by binding to the C-terminus, thereby preventing access of the transferase to its target site^38^. Indeed, starvation induced dissociation of α4 from PP2Ac, leading to PP2A activation and ULK1 dephosphorylation^27^. Methylated PP2Ac cannot interact with the α4 protein, resulting in release of α4 from PP2Ac^20^. Consistently, we confirmed that NNMT KD increases PP2Ac methylation and enhances the enzyme activity (Fig. 4). We also demonstrated that NNMT reintroduction decreased the PP2Ac methylation level, reducing enzyme activity. Collectively, we propose that NNMT KD enhances PP2A methylation and consequently sequesters α4 protein from PP2Ac, leading to PP2A activation and ULK1 dephosphorylation at S638 under nutrient starvation. Although PP2A methylation has been proposed to inhibit autophagy during liver steatosis progression in a mammalian system, the study was conducted under conditions of GNMT KO, which might give rise to severe SAM accumulation^39,40^. Nevertheless, we cannot completely rule out the possibility that PP2A methylation might slow the autophagy process, assuming that the extent of methylation might cause directly opposite results in autophagy regulation. Therefore, whether the degree of PP2A methylation can affect autophagy in different ways is worth further investigation.

Notably, NNMT KD promoted cell survival and growth in vitro and in vivo (Fig. 5a–c). Moreover, NNMT KD reduced necrotic cell death by 2-fold, which is inevitable during tumor growth. We speculate that the beneficial effect might be due to the autophagy acceleration mediated by NNMT KD. To justify our speculation, we treated NNMT KD tumors with autophagy inhibitors. There are numerous inhibitors under development to treat cancer “addicted” to autophagy, such as NSC185058, SAR405, SBI-0206965, chloroquine (CQ), and hydroxychloroquine (HCQ)^41^. Targeting ULK1 could be an effective strategy for inhibiting autophagy in cancer progression because several inhibitors targeting the mid-term or late stages of autophagy could activate pro-survival kinases such as ULK1 during the initial autophagy process^42^. In addition, the only clinically approved autophagy inhibitor is the antimalarial CQ and its derivatives, such as HCQ^15^. Therefore, we applied HCQ or SBI-0206965 (ULK1 inhibitor) to NNMT KD tumors in the mouse model, assuming that an autophagy inhibitor could offset the beneficial effect of NNMT KD on tumor cells under nutrient-deficient conditions. Consistent with our assumption, NNMT KD cancer cells showed resistance to glucose starvation but significant cell death in response to SBI-0206965 treatment as well as HCQ (Fig. 6a, Supplementary Fig. 5). Accordingly, both tumor size and weight decreased in a dose-dependent manner (Fig. 6b–d). In conclusion, these results suggest that autophagy inhibitor treatment could be a feasible strategy for treatment of cancers dependent on NNMT downregulation, typically liver cancers. Likewise, it might be plausible to test NNMT as a marker for companion diagnostics.

In this study, we provided evidence that NNMT could be a negative autophagy regulator in liver cancer. NNMT KD resulted in autophagy induction and accelerated the process under nutrient starvation conditions, while NNMT reversed the phenomenon. We suggest a novel mechanism in which NNMT KD enhances PP2A methylation and activation, leading to ULK1 derepression and autophagy progression (Fig. 6e). Moreover, we showed that NNMT KD predisposed tumor cells to be susceptible to autophagy inhibition, reflecting the beneficial effect of NNMT KD. Given that NNMT KD tumor cells were dependent on autophagy for survival and growth, autophagy inhibitors may be used to treat cancer with NNMT downregulation. Moreover, NNMT expression may be important in companion diagnostics and targeted therapy. The role of NNMT has not been sufficiently elucidated in liver cancer, and thus we clearly need to further investigate NNMT function in liver cancer progression.

## Materials and methods

More detailed reagent information, descriptions of transfections with DNA and siRNAs, and trypan blue, western blot, stable gene KD, and immunohistochemistry assay protocols are provided in the Supplementary Information.

### Cell culture

SK-Hep-1 cells were purchased from American Type Culture Collection (VA, USA), and Hep3B, Huh-7, HepG2, SNU-475, and SNU-449 cell lines were obtained from the Korean Cell Line Bank (Seoul, Korea). The cell lines were tested for mycoplasma contamination (MycoAlert^TM^ Mycoplasma Detection Kit, LONZA, Rockland, ME, USA). Lysates of normal human liver tissue were purchased from Novus (CO, USA). All culture media were obtained from Welgene (Gyeongbuk, Korea). Liver cancer cells were maintained at 37 °C in 5% CO_2_ and cultured in Dulbecco’s Modified Eagle’s Medium or RPMI 1640 supplemented with 10% fetal bovine serum, 100 units/ml of penicillin, and 100 µg/ml streptomycin. Cells were exposed to 10 µM methionine medium for 24 h prior to incubation in Hank’s balanced salt solution (HBSS) for amino acid starvation and in glucose-null or high-glucose medium (as the control) for glucose starvation.

### In vitro methylation assay

The PP2A methylation status was assessed via the NaOH neutralization method^37^. Briefly, 50 µg of lysate was incubated with either preneutralization buffer or base solution to fully demethylate PP2A, followed by neutralization with HCl. Demethylated PP2Ac levels were assessed via western blot analysis. The percentage of methylated PP2A in each sample was calculated by subtracting the demethylated PP2Ac signal in the preneutralization solution without NaOH from the 100% demethylated controls in the base/neutralization solution with NaOH. Normalization was performed using ImageJ software.

### PP2A phosphatase activity

Cells were harvested in lysis buffer and analyzed using a PP2Ac activity assay kit (Upstate, MA, USA), according to the manufacturer’s instructions. The control in each experiment was vehicle treatment of negative control cells, for which the value was set to 1.0.

### Xenograft tumorigenesis

Five-week-old female BALB/c nude mice were obtained from Orient Bio (Gyeonggi, Korea). The mice were subcutaneously injected with SK-Hep-1 cells (5 × 10^6^) transfected with the negative control (N.C.) or with NNMT shRNA in the right and left abdominal areas, respectively. Tumor sizes were measured once a week. The mice were sacrificed 8 weeks after injection, and their solid tumors were isolated. To assess the effect of SBI-0206965 on tumor growth, SK-Hep-1 cells (1 × 10^7^) were injected subcutaneously. The tumors were allowed to grow for 14 days, and selected according to the tumor size (>50 mm^3^). The selected mice were randomized blindly (*n* = 5 for each treatment group). SBI-0206965 (0, 5, or 20 mg/kg for each group) was intraperitoneally injected three times a week. The tumor volumes were determined by the formula *W*^2^ × *L* × 1/2, where *L* is the length and *W* the width of the tumor. All animal experiments were performed in accordance with the relevant guidelines and regulations and with the approval of the Institutional Animal Care and Use Committees at POSTECH. This study did not involve human participants, and thus participation consent was not applicable.

### Immunofluorescence microscopy

Cells were transiently transfected with plasmid encoding ptfLC3 (Addgene, MA, USA). After 36 h of transfection, the cells were incubated with HBSS for 4 h. After a PBS wash, the cells were fixed with 3.7% paraformaldehyde for 20 min at RT and then washed twice with PBS prior to mounting with coverslips on glass slides. Fluorescence images were obtained using a confocal microscope (Leica, Wetzlar, Germany). The number of LC3 puncta per cell was determined triplicates by counting a total of more than 30 cells, and the signal intensity per cell was measured and quantified with ImageJ software.

### Statistical analysis

All samples but animals were prepared as triplicates for every experiment. The data represent at least three independent experiments and are presented as the mean ± standard deviation. The data are presented as bar graphs and were statistically verified with Student’s *t*-tests. The *p*-values < 0.05 were considered statistically significant and are indicated with a single asterisk (*), two asterisks (**), or three asterisks (***) for *p*-values < 0.05, <0.01, or <0.001, respectively.

## Electronic supplementary material

Supplementary figure_1

Supplementary figure_2

Supplementary figure_3

Supplementary figure_4

Supplementary figure_5

Supplementary Information
