# Supplementary figures and images for: NNMT depletion contributes to liver cancer cell survival by enhancing autophagy under nutrient starvation
Source: Oncogenesis. 2018 Aug 10;7(8):58. doi: 10.1038/s41389-018-0064-4 (PMC6085294; doi:10.1038/s41389-018-0064-4)

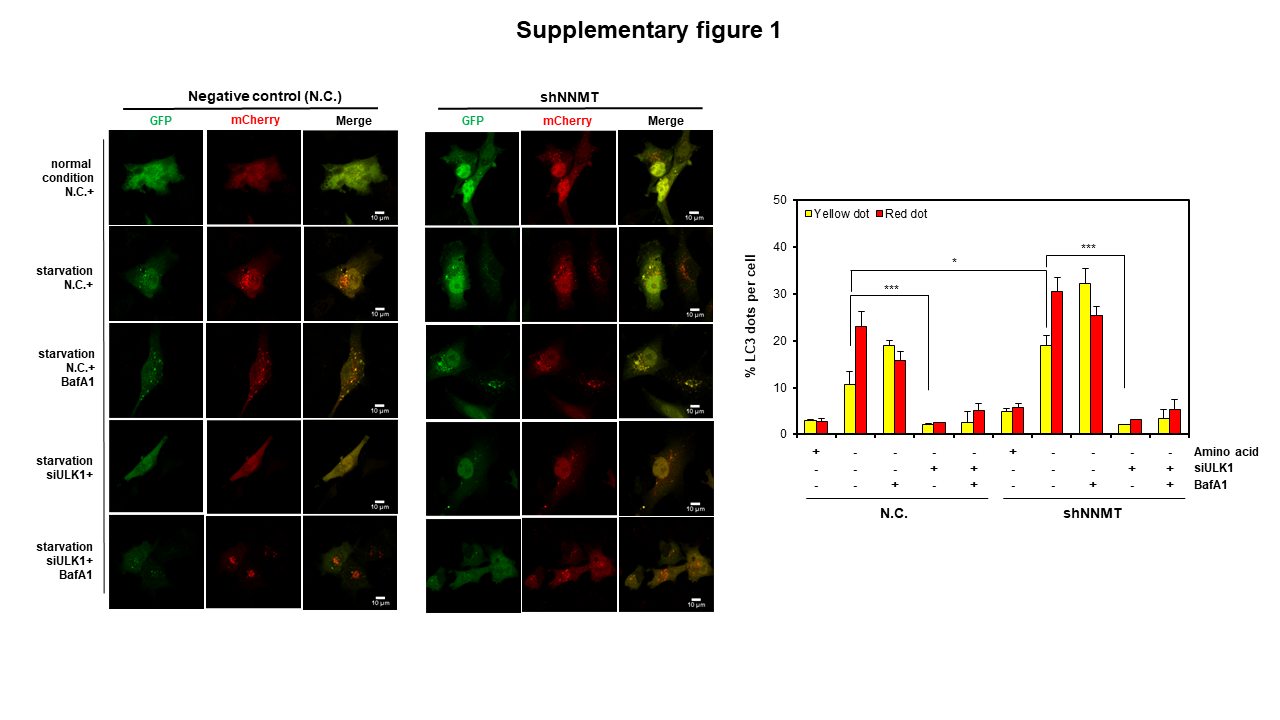

Supplement: Supplementary file 1 — Supplementary figure_1 [file 41389_2018_64_MOESM1_ESM.tif]

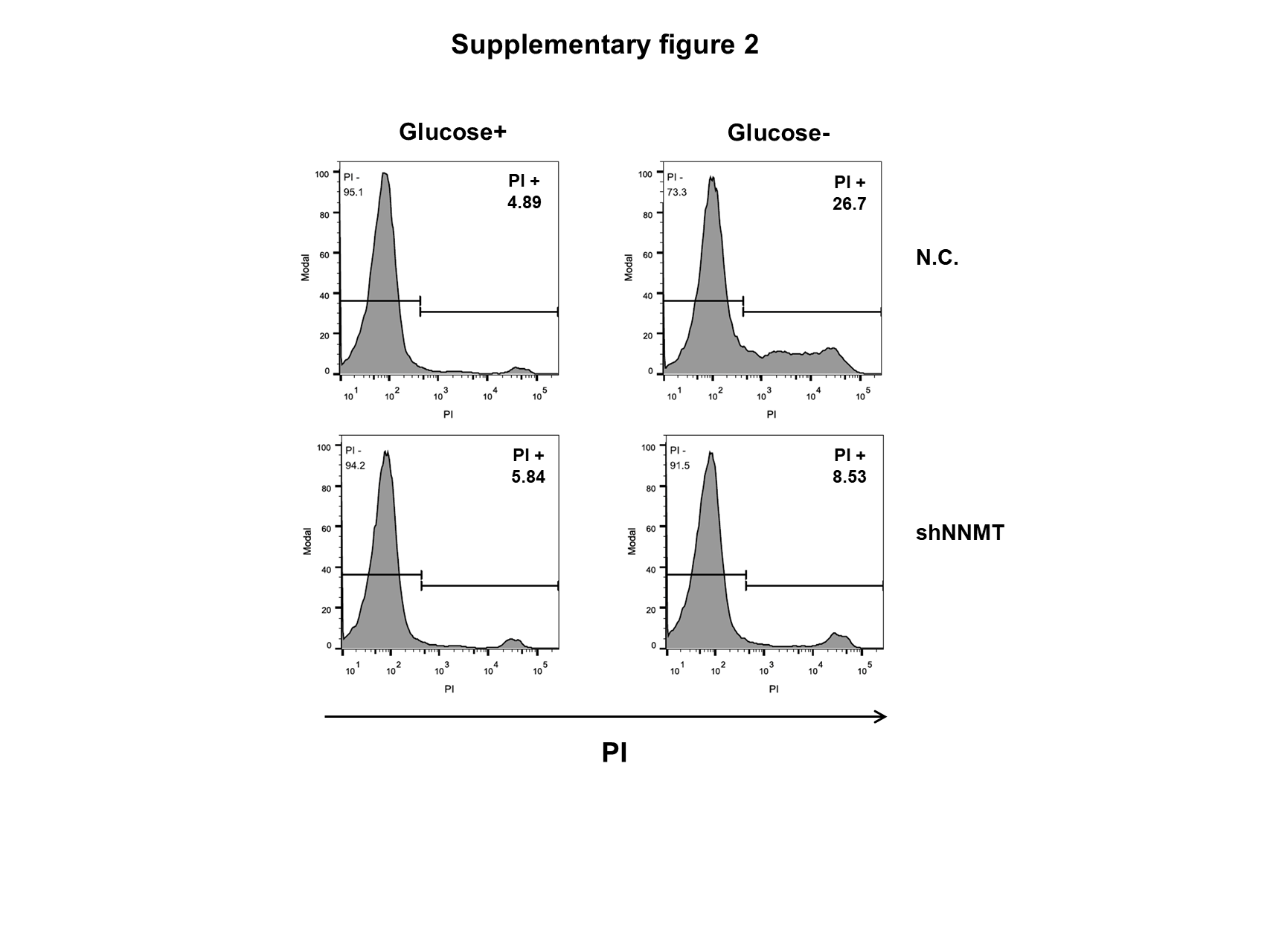

Supplement: Supplementary file 2 — Supplementary figure_2 [file 41389_2018_64_MOESM2_ESM.tif]

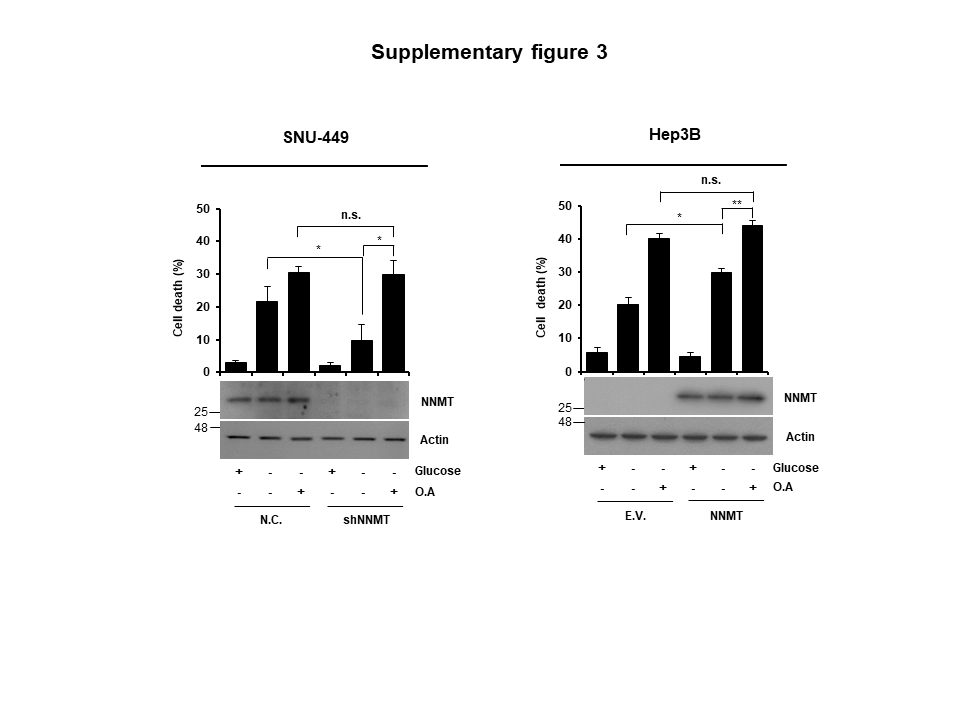

Supplement: Supplementary file 3 — Supplementary figure_3 [file 41389_2018_64_MOESM3_ESM.tif]

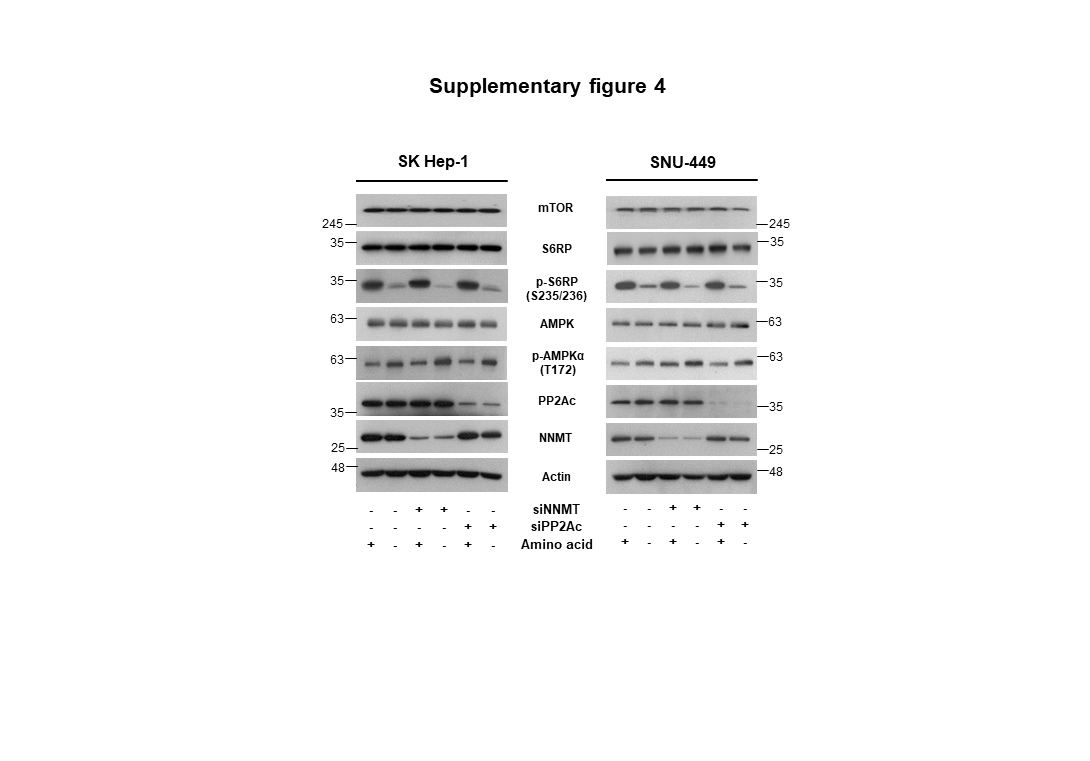

Supplement: Supplementary file 4 — Supplementary figure_4 [file 41389_2018_64_MOESM4_ESM.tif]

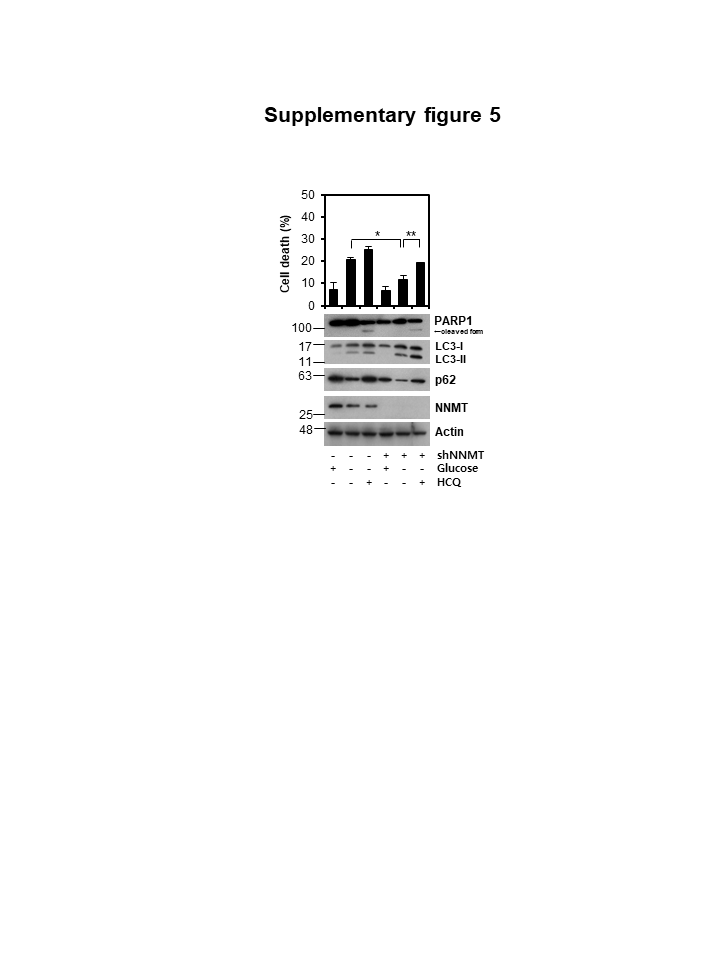

Supplement: Supplementary file 5 — Supplementary figure_5 [file 41389_2018_64_MOESM5_ESM.tif]
