# Supplementary material for: NNMT depletion contributes to liver cancer cell survival by enhancing autophagy under nutrient starvation
Source: Oncogenesis. 2018 Aug 10;7(8):58. doi: 10.1038/s41389-018-0064-4 (PMC6085294; doi:10.1038/s41389-018-0064-4)
Supplement: Supplementary file 6 — Supplementary Information [file 41389_2018_64_MOESM6_ESM.doc]

**Supplementary Figure legends**

**Fig S1. ULK1 inhibition blocks autophagosome formation irrespective of NNMT expression levels.** SK-Hep-1-N.C. or –shNNMT cells were transiently transfected with either empty vector (E.V.) or green fluorescent protein (GFP)-mCherry tandem fluorescence-tagged LC3 plasmid for 20 h and exposed to amino acid starvation for an additional 4 h with or without 10 nM BafA1 treatment. Confocal microscopy images for autophagosome analysis. Scale bar indicates 10 µm. The histogram represents the % LC3 puncta per cell, with yellow (autophagosome). The data are shown as the means ± SD (**p*<0.05, ****p*<0.001)

**Fig S2. NNMT knockdown cells confer resistance to nutrient deprivation.** Cell death in SNU-449-N.C. or –shNNMT was assessed by flow cytometry using PI staining. Cells were incubated in glucose deprivation for 60 h. Acquired data were analyzed using the FlowJo software.

**Fig S3. Okadaic acid increases sensitivity to nutrient starvation in NNMT knockdown cells.** SNU-449-N.C. or –shNNMT cells were incubated with high glucose or glucose-null medium for 24 h, followed by with or without okadaic acid treatment for additional 24 h. Hep3B cells E.V. or -NNMT OE cells were subjected to glucose starvation for 30 h, followed by in the presence or absence of okadaic acid treatment for additional 18 h. Cell death was assessed with trypanblue assay. Each histogram bar represents the means ± SD of three independent experiments (**p*<0.05, ***p*<0.01, n.s., not significant)

**Fig S4. mTORC1 or AMPK is not affected by PP2Ac knockdown under nutrient starvation.** Levels of the indicated proteins were analyzed by western blot in SK-Hep-1 and SNU-449 cells. Cells were transfected non-targeting (negative control, N.C.) or NNMT or PP2Ac siRNAs for 44 h, followed by amino acid starvation for an additional 4 h.

**Fig S5. NNMT knockdown protects liver cancer cells from nutrient starvation via autophagy.** SNU-449-N.C. or -shNNMT cells were cultured in glucose starvation medium for 12 h and treated with 10 µM HCQ for an additional 24 h. Cell death was assessed with a trypan blue assay, and western blot analysis of autophagic markers was performed in cell lysates. All data from the blots are representative of three independent experiments, and each histogram bar represents the means ± SD (**p*<0.05, ***p*<0.01).

**Supplementary Information**

**Chemical reagents**

Bafilomycin A1 and rapamycin were obtained from Sigma Aldrich (MO, USA). FTY 720, hydroxychloroquine and SBI-0206965 were purchased from Cayman Chemical (MI, USA). Okadaic acid was purchased from Enzo Life Sciences (NY, USA).

**Transient transfection with DNA and siRNA**

Negative control siRNA, On-TARGETplus SMARTpool human NAMPT siRNA (Cat. No. M004581-01-0005), On-TARGETplus SMARTpool human ULK1 siRNA (Cat. No. L005049-00-0005), On-TARGETplus SMARTpool human PP2Ac siRNA (Cat. No. L003598-01-0005) and On-TARGETplus SMARTpool human NNMT siRNA (Cat. No. L-010351-00-0005) were purchased from Dharmacon. siRNA duplexes were added at a final concentration of 100 nM. To generate the NNMT-rescue construct expressing NNMT mRNA resistant to the NNMT short hairpin RNA, site directed mutagenesis was conducted. Synonymous mutations (depicted with uppercase letters) were introduced into the NNMT shRNA target sequence (5’-attctgcctGgacggAgtgaa-3’) within the NNMT coding-region of the FLAG-tagged NNMT plasmid (Origene, RC200641) via polymerase chain reaction. Sequencing of the complementary DNA (cDNA) was performed to confirm the mutation. Five hours after transfection with siRNA or the plasmid using Lipofectamine (CA, USA), transfected cells were cultured in growth medium for 72 h.

**Lentiviral infection to construct stable cell lines**

Lentiviral particles expressing NNMT shRNA targeting NNMT (NM_006169) and non-targeting shRNA were purchased from Sigma Aldrich (MO, USA). Cancer cells were transfected with the lentiviral particles using 8 µg/ml polybrene. After 24 h, cells were selected with 4 µg/ml of puromycin for one month. For establishment of stable NNMT overexpression cell lines, Precision LentiORF Ploc lentiviral vectors expressing NNMT and a control were purchased from Open Biosystems (CO, USA). Transfected cells were selected with 8 µg/ml of blasticidine for one month.

**Trypan blue assay**

Cells were harvested, washed with PBS and then treated with trypan blue reagent in PBS for 5 min at room temperature (RT). At least 200 cells were counted per assay using a hemocytometer. The percentage of dead cells was determined by counting the number of blue cells in a population.

**Flow cytometry**

Cells were trypsinized for harvest and then washed with cold PBS. Harvested cells counted and resuspended at a concentration of 2x10^5 cells/100ul. Suspended cells were treated with propidium iodide (PI) staining reagents (BD Biosciences), and then incubated for 20 min at RT in a dark prior to the analysis by a flow cytometer (BD Biosciences, Canto II). Acquired data were analyzed using the FlowJo software.

**Western blot analysis**

Immunoblotting was performed as described previously (43). Primary antibodies targeting the following proteins were used: NNMT (Abcam, UK), FLAG, Actin (Sigma, MO, USA), PP2A, methyl-PP2A, and demethyl-PP2A (Millipore, MA, USA), p62 (Santa Cruz, TX, USA), LC3B, phosphoSer758-ULK1, phosphoSer638-ULK1, phosphoSer555-ULK1, ULK1, ATG7, Beclin-1, phosphoThr172-AMPKα, AMPKα, mTOR, phosphoSer235/236-S6RP, S6RP and PARP1 (Cell Signaling Technology, MA, USA).

**Immunohistochemistry**

For these assays, 5-mm tissue slices of xenograft tumors were prepared, fixed and embedded in paraffin. The tissues were subjected to Ki-67 (Santa Cruz, TX, USA) staining to visualize proliferating cells. The slices were counterstained with hematoxylin and eosin. The regions of cell death were assessed using a terminal deoxynucleotidyl transferase (TdT) dUTP nick-end labeling (TUNEL) assay kit (Trevigen, MD, USA) according to the manufacturer’s instruction. Stained slices were scanned at 10X with a virtual microscope scanner (Olympus). All images of the tumor sections were captured and analyzed with ImageJ software.
